# On-Surface Synthesis and Cryogenic Exfoliation of Sterically Frustrated Atropisomers

**DOI:** 10.1021/acsnano.4c16645

**Published:** 2025-04-01

**Authors:** Philipp D’Astolfo, J.G. Vilhena, Simon Rothenbühler, Carl Drechsel, Oscar Gutiérrez-Varela, Robert Häner, Silvio Decurtins, Shi-Xia Liu, Giacomo Prampolini, Rémy Pawlak, Ernst Meyer

**Affiliations:** † Department of Physics, University of Basel, Klingelbergstrasse 82, 4056 Basel, Switzerland; ‡ CSIC, Instituto de Ciencia de Materiales de Madrid (ICMM), 28049 Madrid, Spain; § Department of Chemistry, Biochemistry and Pharmaceutical Sciences, W. Inäbnit Laboratory for Molecular Quantum Materials, University of Bern, Freiestrasse 3, CH 3012 Bern, Switzerland; ∥ CNR−Consiglio Nazionale delle Ricerche, Istituto di Chimica dei Composti Organo Metallici (ICCOM-CNR), Area della Ricerca, via G. Moruzzi 1, I-56124 Pisa, Italy

**Keywords:** on-surface synthesis, stereochemistry, nanomanipulation, molecular dynamics, SPM

## Abstract

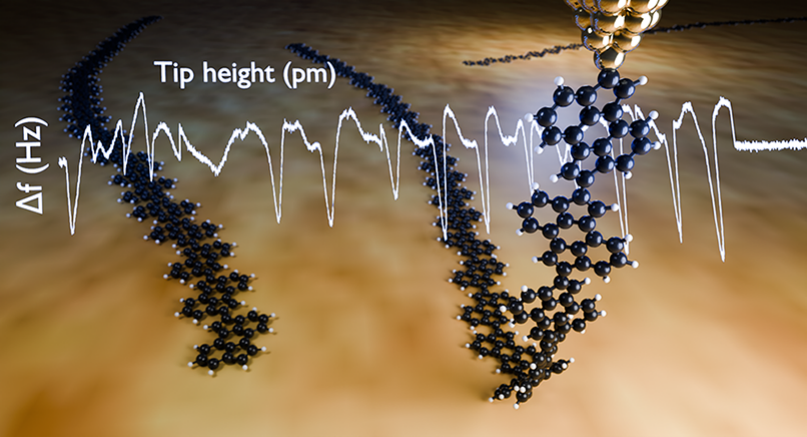

On-surface synthesis provides exceptional control over nanostructure and material composition, enabling the creation of molecular compounds that are difficult or impossible to obtain with other synthesis methods. In this work, we demonstrate the possibility of synthesizing atropisomeric molecules made of chains of polyaromatic hydrocarbon units via on-surface synthesis. Scanning probe microscopy reveals that molecules adsorbed on Au(111) surfaces adopt a planar structure, with adjacent monomeric units aligning either in parallel or antiparallel configurations, influencing the alignment of the molecule on the surface. Cryo-force spectroscopy peeling experiments show that metastable conformers can be mechanically stabilized during the lifting-redeposition process of the polymer from the surface. In this process, periodic drops in frequency shift are observed, corresponding to monomer detachment-readsorption. Interestingly, this periodicity is independent of the parallel/antiparallel configuration but is counterintuitively smaller than the monomer size. Molecular dynamics simulations relate this effective reduction in unit length to a tethering effect between the chain and the surface. This, in turn, allowed us to test and validate Silva’s analytical phenomenological power law model for peeling. Our findings not only provide a method for studying the elusive class 1 atropisomeric molecules but also offer deeper insight into the peeling phenomenon at the nanoscale.

Over the past decade, on-surface synthesis has been the subject of vibrant research studies.^1−9^ Empowered by advances in surface science techniques^10−14^ and theoretical calculations,^14−21^ a wealth of reactions have been performed on a variety of surfaces, and most notoriously, the reaction mechanisms have been scrutinized—some for the first time—with submolecular resolution imaging of the products as well as intermediate states.^8,22,23^ Complementary to supramolecular chemistry, where large molecules are held together by weak interactions, on-surface synthesis allows for a bottom-up design of functional nanoarchitectures^24^ held together by strong covalent interactions, thus being able to withstand harsher conditions.^4−7,25^ Besides the versatility of a bottom-up design of covalent bonds between individual molecular building blocks, on-surface synthesis is also imbued with an atomically precise control of its products, a feature of paramount importance and often unmet in competing top-down approaches.^4,26−28^ The new materials thus discovered have shown great potential in various applications, including electronic devices,^29^ spintronic devices,^30^ highly efficient catalysts,^31^ and the control/design of stable chiral structures.^32^

One peculiarity of on-surface synthesis is that the surface interaction acts as a 2D restraint on the reactants. As a result, nonplanar aromatic compounds are forced to remain flat as the adsorption energy competes—and often overcomes—steric hindrance, leading to planarity.^19,33^ A simple yet exegetical example of such a mechanism has been reported for biphenyl, where the dihedral angle ruling the coplanarity of the two aromatic rings is found at ∼40° in the gas phase, whereas it remains flat when stacking *pi–pi* interactions are settled with the surrounding environment.^34−38^ The situation becomes more interesting when torsion is asymmetric as in a biphenyl where an ortho-hydrogen is replaced by fluorine—resulting in two distinct flat conformations: one with two facing fluorines and another with a fluorine facing a hydrogen atom.^39,40^ The first conformer is less stable, owing to the electronic repulsion between the fluorides. Thus, through the 2D confinement, one may stabilize conformations that are otherwise rarely sampled or even inaccessible in the gas phase. This property is of particular interest in stabilizing atropisomers—molecules possessing a hindered torsional rotation about the aryl–aryl single bond, with such a high energy barrier that distinct conformers may be stabilized.^41,42^ Atropisomerism may give rise to geometrical isomers, diastereoisomers, or enantiomers. Although technical advances in chiral separation and stereochemically controlled synthesis allow detailed control of chiral drugs,^43−47^ which otherwise may have dramatic consequences, e.g., thalidomide, there is no established pharmaceutical protocol for atropisomers, whose lifetimes can spawn from minutes to years.^48^ In this context, on-surface chemistry offers a route to synthesize such atropisomers.

Beyond structural characterization, scanning probe microscopy (SPM) also provides insights into the nonequilibrium properties of molecules synthesized in this way. In particular, cryo-force-spectroscopy^12,14,33^ provides a valuable means for exploring the mechanical and tribological properties of molecules in ultrahigh vacuum (UHV) conditions at low temperatures (LT). Such a controlled environment allows us to understand how internal degrees of freedom of a given molecule participate in different dissipative and mechanical processes.^8,12−14,17,19,33^ However, to date, most studies concerned about the manipulation of achiral molecules that, upon desorption, may only adopt a given conformation. Also, it is unclear if cryo-force spectroscopy could be harnessed to mechanically stabilize different stereoisomers—a feature of utmost interest for nanoconfined light–matter interactions.^49,50^

In this work, we investigate polyaromatic hydrocarbon (PAH) molecular chains belonging to class 1 atropoisomerism as described by Kemp et al.^51^ By combining cryo-force spectroscopy and all-atom molecular dynamics (MD) simulations with quantum-mechanically derived force-fields (QMD-FF),^52,53^ we explore the effects of 2D confinement on stereochemical control, mechanical stability, and peeling dynamics. Unlike their gas-phase counterparts, the molecules adopt a planar conformation on the Au(111) surface due to 2D confinement. This results in contiguous monomers adopting either parallel or antiparallel arrangements. Despite the significant steric repulsion associated with the antiparallel configuration, our experiments reveal a racemic mixture of these conformations. This finding highlights the role of 2D confinement in stabilizing sterically frustrated states that would otherwise be inaccessible in the gas phase. Furthermore, we find that the stereochemical conformation of the polymers strongly influences their alignment on the Au(111) surface, leading to a rich variety of on-surface configurations. Cryo-force spectroscopy and MD simulations demonstrate, for the first time, the possibility of mechanically stabilizing metastable conformers by desorbing them from the surface. Interestingly, such peeling experiments also show that the detachment length is consistently shorter than the monomer size, a finding that differs from previous studies on the exfoliation of PAH molecules.^13,33^ MD simulations attribute this behavior to tethering effects caused by interactions with surface defects and other polymers. These findings allow the distinction by the first time of two distinct peeling mechanisms: (i) sliding exfoliation and (ii) delamination. Finally, we validate the analytical peeling model proposed by Silva et al.,^15^ demonstrating its broader applicability.

## Results and Discussion

### On-Surface Synthesis of Sterically Frustrated Polymers

**Figure 1:**
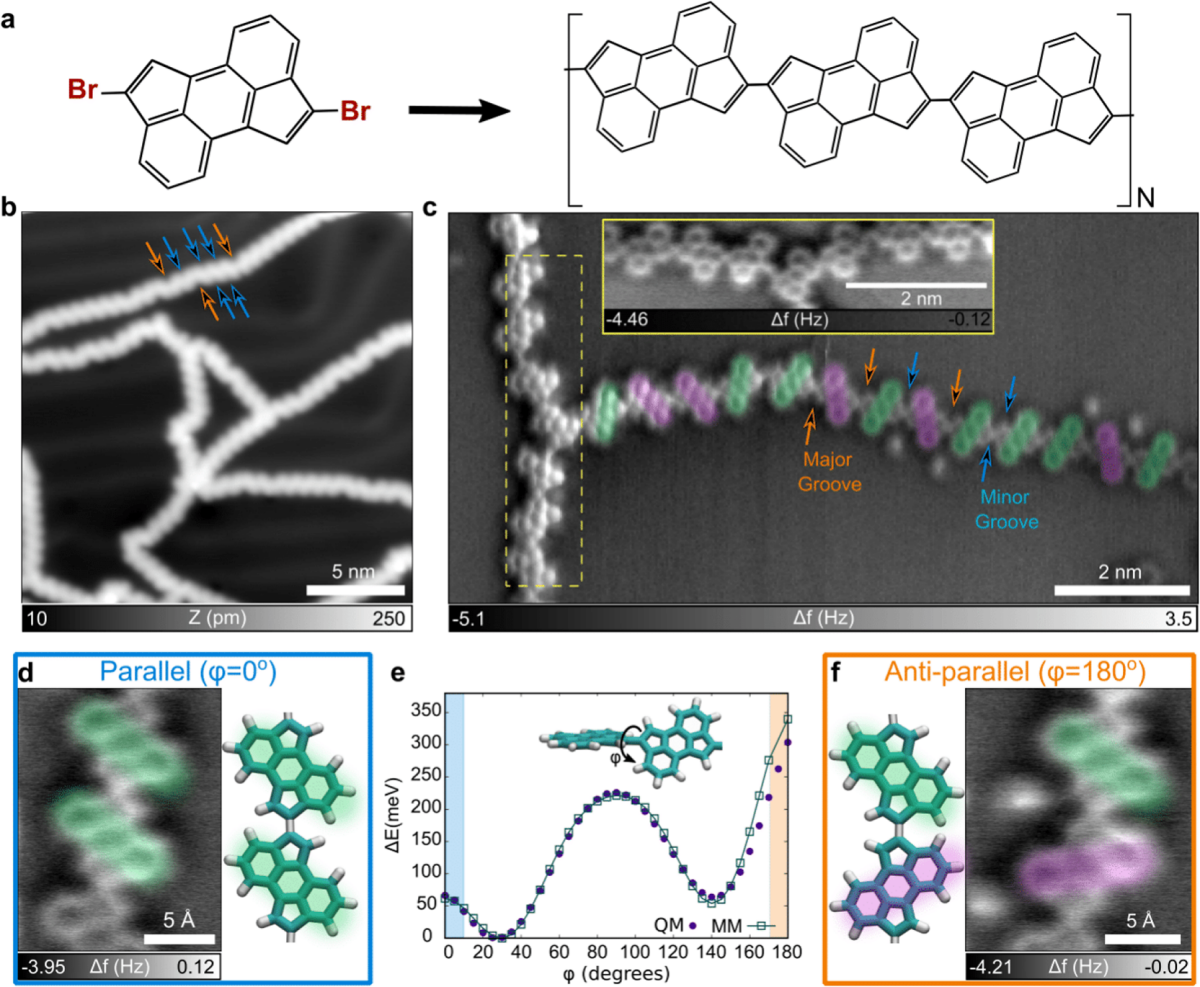
On-surface synthesis of a class 1 atropisomer. (a) Chemical structure of 2,7-dibromocyclopent[*h,i*]aceanthrylene (2,7-dibromo-CPAA). (b) STM image of interlinked CPAA polymers (*I*_t_ = 1 pA, *V*_s_ = 150 mV). (c) AFM image was measured with a CO-terminated tip of a CPAA polymer with parallel and alternating bond conformation. The arrows in (b) and (c) indicate the different grooves associated with parallel arrangements (blue arrows) or antiparallel arrangements (orange arrows) of contiguous CPAA monomers. (d and f) Detailed view of parallel (antiparallel) bonded units. (e) Energy barriers obtained by rotation of the aryl–aryl bond between two units using QMD-FF based (MM) and DFT (QM) calculations as detailed in Methods.

Purposely synthesized 2,7-dibromocyclopent[h,i]aceanthrylene monomers (2,7-dibromo-CPAA, as shown in Figure 1a) are evaporated onto a clean Au(111) surface and subsequently polymerized via Ullmann coupling.^4,19,33^ These polymers are then imaged using a combined scanning tunneling microscope (STM) and atomic force microscopy (AFM) operated at a low temperature (4.8 K) under UHV. Further details on synthesis and imaging are provided in Methods.

Figure 1b,c represents STM and AFM images providing an atomically detailed understanding of CPAA polymerization. In particular, the most salient feature is that CPAA predominantly polymerizes in a linear fashion along the para-axis, whereby new C–C bonds are formed between consecutive monomers. However, occasionally a single CPAA monomer connects to a third monomer sideways via a dehydrogenation process. Such events result in a sparse cross-linked network between these otherwise linear chains. Interestingly, Urgel et al. also reported the on-surface polymerization of CPAA polymers.^54^ However, their synthesis involved significantly higher annealing temperatures, favoring cyclodehydrogenation between consecutive monomers and yielding structures differing from those considered here. Despite these differences, in both cases, the CPAA monomers remain flat on Au(111), as seen in Figure 1b,c. This gives rise to a high steric repulsion between hydrogen atoms of neighboring monomers, as shown by our first-principles calculations (see Figure 1d–f). At the surface, consecutive monomers adopt a torsion angle of ϕ = 0° or 180°, whereas in solution or the gas phase, the angle is always ϕ ∼ 30°. Consequently, both parallel or antiparallel configurations between monomers (Figure 1d,f) are obtained at the surfaces, which is in stark contrast with solution synthesis. Thus, the on-surface reaction enables the stabilization of atropisomers at surfaces. However, the synthetic approach of our system is also accompanied by a cyclodehydrogenation process, which induces uncontrolled cross-linking between the polymeric chains.

Figure 1b illustrates an uneven spacing between consecutive monomers in the form of major and minor grooves along the chain, indicated by orange and blue arrows. The AFM image in Figure 1c provides evidence that these grooves result from variations in the arrangement of consecutive monomers, where ϕ takes values of either 0° or 180°. Specifically, when consecutive units are parallel with ϕ = 0°, as depicted in Figure 1d, the spacing between consecutive units is symmetric with respect to the long axis of the polymer, resulting in a minor groove. However, when consecutive units are antiparallel with ϕ = 180°, as shown in Figure 1f, the spacing between consecutive units becomes asymmetric with respect to the long axis of the polymer. This leads to a significant difference in the distance between neighboring hydrogen atoms on one side of the chain compared with the other, resulting in a major groove. Surprisingly, although the antiparallel conformation is associated with much higher steric repulsion between neighboring units, with an associated energy of Δ*E*^anti^ ∼ 300 meV according to our DFT calculations (shown in Figure 1e; see Methods), both conformations appear in a racemic mixture, as shown in Figure 1c. The 2D on-surface confinement provides the means to catalyze the synthesis of sterically frustrated conformers, otherwise inaccessible in both gas and liquid phases, as the required energies are in the range of 3000 K.

### Isomeric-Dependent Adsorption Conformation

**Figure 2:**
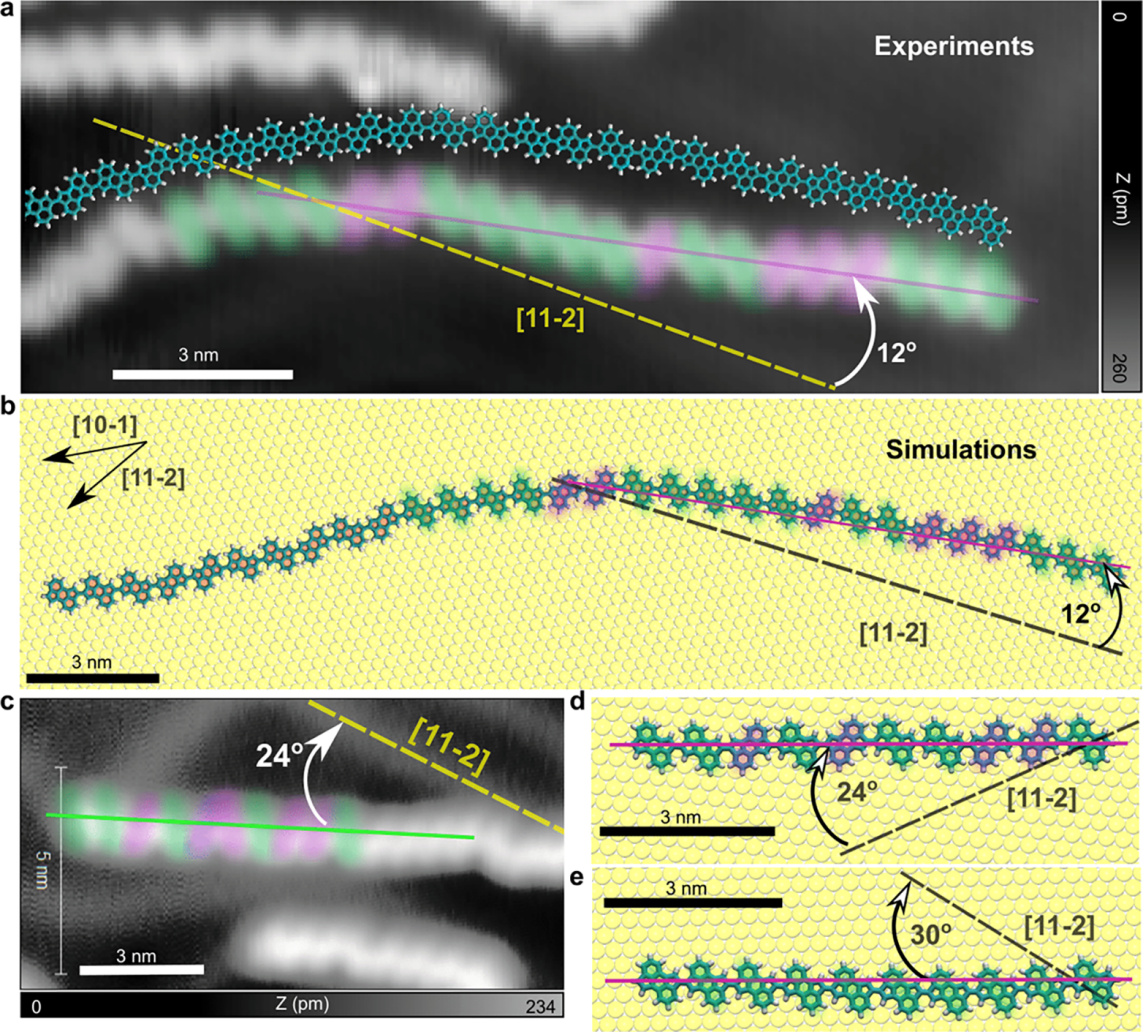
Adsorption geometries of CPAA on Au(111) and its dependence on the relative units orientation. (a) STM image of CPAA on Au(111) after quenching from 450 to 4.8 K (*I*_t_ = 1 pA, *V*_s_ = −80 mV). An MD atomic representation of the chain is overlaid. The purple and yellow lines depict the angle between the chain and the crystallographic direction [11–2] (parallel to the herringbone). (b) Adsorption configuration of a 30-mer CPAA modeled using MD (see Methods). The relative orientation of the monomers is highlighted in green and purple, as in (a). In the top left corner, the compact direction [10–1] and the noncompact direction [11–2] are indicated. (c) STM image of the adsorption configuration of another CPAA chain. Note the different parallel/antiparallel unit arrangement as compared to that of (a) now results in a 24° angle with respect to [11–2]. (d and e) MD equilibrium adsorption configuration of a 10-mer CPAA chain obtained using (d) a units arrangement similar to that shown in (c); and (e) arrangement of units completely parallel to each other.

The Au(111) surface, with its hexagonal atomic arrangement, exhibits two main crystallographic directions: the noncompact [11–2] direction and the compact [10–1] direction (see Figure 2b), respectively corresponding to a less or more densely packed atomic structure. Throughout this work, these Miller indices are consistently used to refer to these crystallographic directions. Figure 2a shows a long CPAA polymer adsorbed over a Au(111) surface, as imaged using STM (see Methods). The long axis of the polymer (depicted by a purple line) forms an angle of 12° with respect to the direction [11–2], or equivalently, an angle of 18° with respect to the direction [10–1]. Therefore, and in contrast with prior works^33^ on similar PAH chains on Au(111), CPAA chains do not align along any specific crystallographic direction of Au(111).

To gain an atomically detailed understanding, we performed all-atom molecular dynamics simulations of the CPAA adsorption process, as detailed in Methods. To ensure an accurate description of the interatomic interactions, a QMD-FF was purposely derived as explained in Methods and Section S1 of the Supporting Information. The simulation result obtained for an equivalent chain to Figure 2a, i.e., with similar length and monomer relative orientations, is shown in Figure 2b. As in the experiments, MD simulations predict an equilibrium adsorption configuration in which the long axis of the polymer forms an angle of 12° with respect to the [11–2] direction. Such reproducibility suggests that the noncrystallographic orientation shown in Figure 2a,b is not random. It should be noted that to prevent an initial bias in our simulations, we annealed the system up to 450 K. At such high temperatures, the simulations reveal a rapid diffusion of CPAA polymers, thus sampling many relative orientations over the Au(111) surface. Subsequently, we quenched the temperature from 450 to 5 K in 5 ns, a period during which the polymer aligns into a given low-temperature equilibrium configuration, as shown in Figure 2b. The atomically detailed image of the entire quenching process is provided in Supporting Movie S1.

Concomitantly, experiments show that different arrangements of the contiguous CPAA units result in different alignments of the polymer long axis with respect to the [11–2] crystallographic direction. In particular, Figure 2c shows a CPAA chain at a 24° angle with respect to the [11–2] direction, at variance with the polymer illustrated in Figure 2a,b. Interestingly, the major difference between these two chains is the relative orientation of consecutive monomers, as highlighted by green or purple ellipses in Figure 2a,c. By conducting MD simulations with the relative orientation of the monomers adjusted to match the second chain (i.e., Figure 2c), we obtain an equilibrium adsorption configuration in which the CPAA long axis forms an angle of 24° with [11–2], as illustrated in Figure 2d—in excellent agreement with the experimental findings in Figure 2c. Thus, altering the relative orientation between units results in a change of the adsorption configuration. This is also corroborated in Figures S2 and S3 in Section S2 of the Supporting Information for different arrangements in MD simulations. Lastly, we repeated the simulations for a third conformer (i.e., a conformational isomer) with all monomers arranged in a parallel fashion. The resulting equilibrium adsorption configuration, shown in Figure 2e, is at an angle of 30° with [11–2], or equivalently, perfectly aligned to the [10–1] crystallographic direction, identical to the graphene ribbon adsorption configuration.^12^

Compared with previous works on PAH chains on Au(111), our findings reveal a novel connection between the relative orientation of consecutive monomers (i.e., conformation-isomerism) and the adsorption configuration of the molecule on the surface. Prior reports consistently show that PAH chains align along one of two high-symmetry crystallographic directions of Au(111), i.e., either along the direction [10–1] or [11–2]. In contrast, nearly every CPAA polymer chain has a different orientation angle, with respect to the underlying Au(111) substrate. While further investigation is needed, our results suggest that the orientation angle with respect to the surface crystallographic directions is unique to each conformational isomer of the chain. The implications of this finding are wide-ranging. For example, it means that for a given molecule/contact pair, simply by altering the orientation between consecutive monomers, we can alter its registry with the surface and, thus, its friction. Therefore, it is expected that the friction experienced by this molecule when sliding on a metallic surface is not uniquely defined, something that will be addressed in future works. Additionally, this coupling between surface orientation and conformational isomerism could be exploited to select different stereochemical conformers, which could have implications for a range of applications such as molecular electronics and catalysis.

### Cryogenic Exfoliation of Metastable Class 1 Atropisomeric Chains

**Figure 3:**
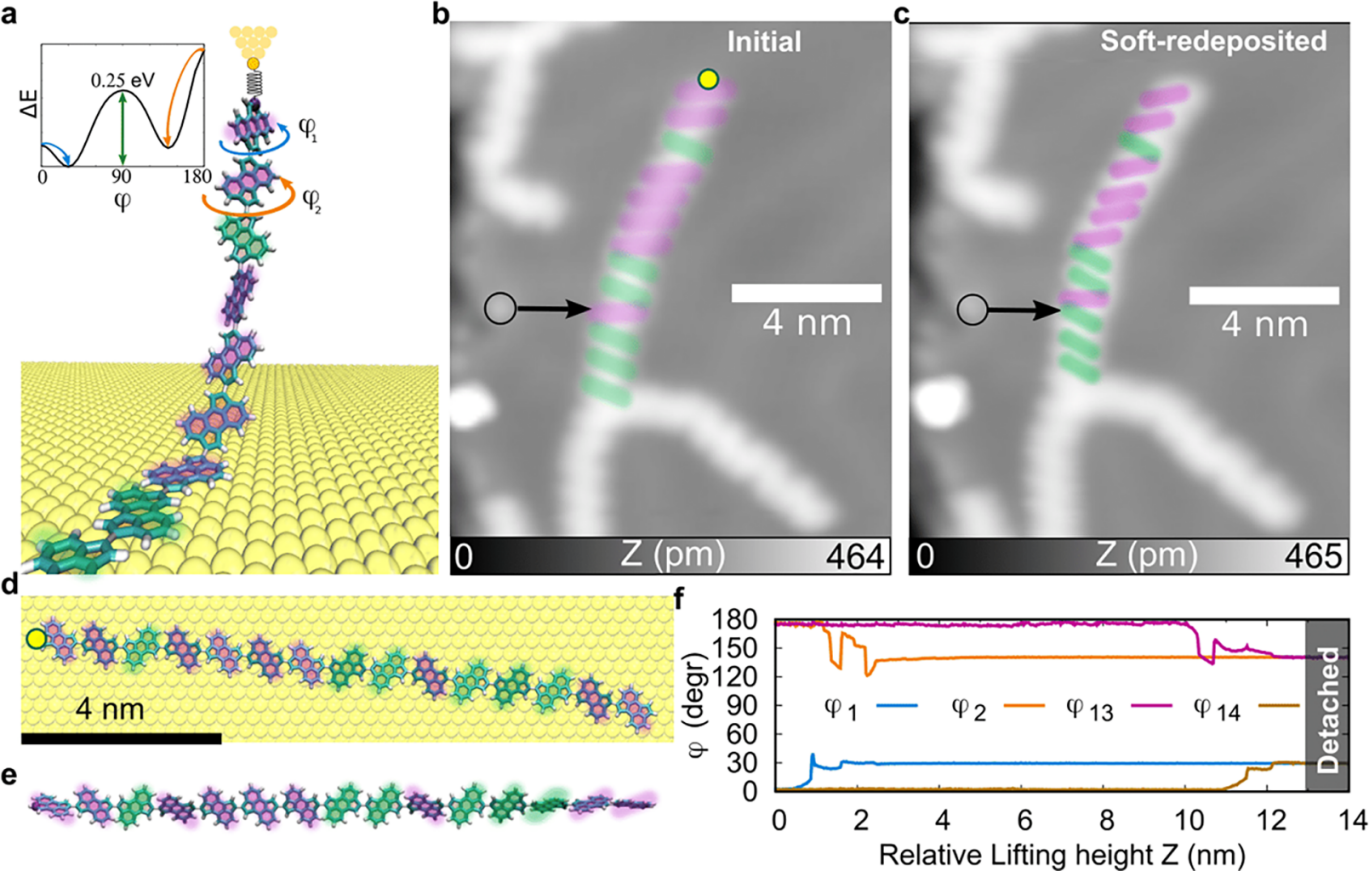
Sterically frustrated CPAA conformers: desorption stability. (a) MD snapshot of the desorption process, highlighting the torsion angles of the first two units after detachment. The inset graph corresponds to the energy barriers obtained by rotation of the aryl–aryl bond between the two units (shown in Figure 1e). (b) and (c) (*I*_t_ = 1 pA, *V*_s_ = 100 mV) before and after manipulation, with the black arrow marking the same region, showing slight chain displacement. (d) MD configuration of the chain on the surface before manipulation and (e) its configuration after manipulation and complete detachment from the surface. Note how the conformations between monomers are the same in both cases. The yellow dots in parts (b) and (d) mark where the tip contacts the chain to form the tip-chain bond. (f) MD torsion angles during desorption, showing stable dihedrals after detachment, highlighting the molecule’s structural stability.

To investigate whether these sterically frustrated polymers were stable upon desorption, we resorted to cryo-force spectroscopy,^8^ as shown in Figure 3a. By pressing the AFM tip against a CPAA-chain termination (Figure 3b) a stable tip-molecule bond is formed, which allow us to lift the molecule from the surface by lifting the AFM tip (see Methods for further^8,12,13,33^ details). Subsequently, the molecule is redeposited back onto the surface (Figure 3c) by lowering the AFM tip. Furthermore, an atomically detailed picture of this process is provided by all-atom molecular dynamics (MD) simulations with purposely tailored QMD-FF (see Methods). These simulations carefully match the experiments, specifically the parallel/antiparallel motif between consecutive monomers prior to its desorption (see Figure 3b,d).

**Figure 4:**
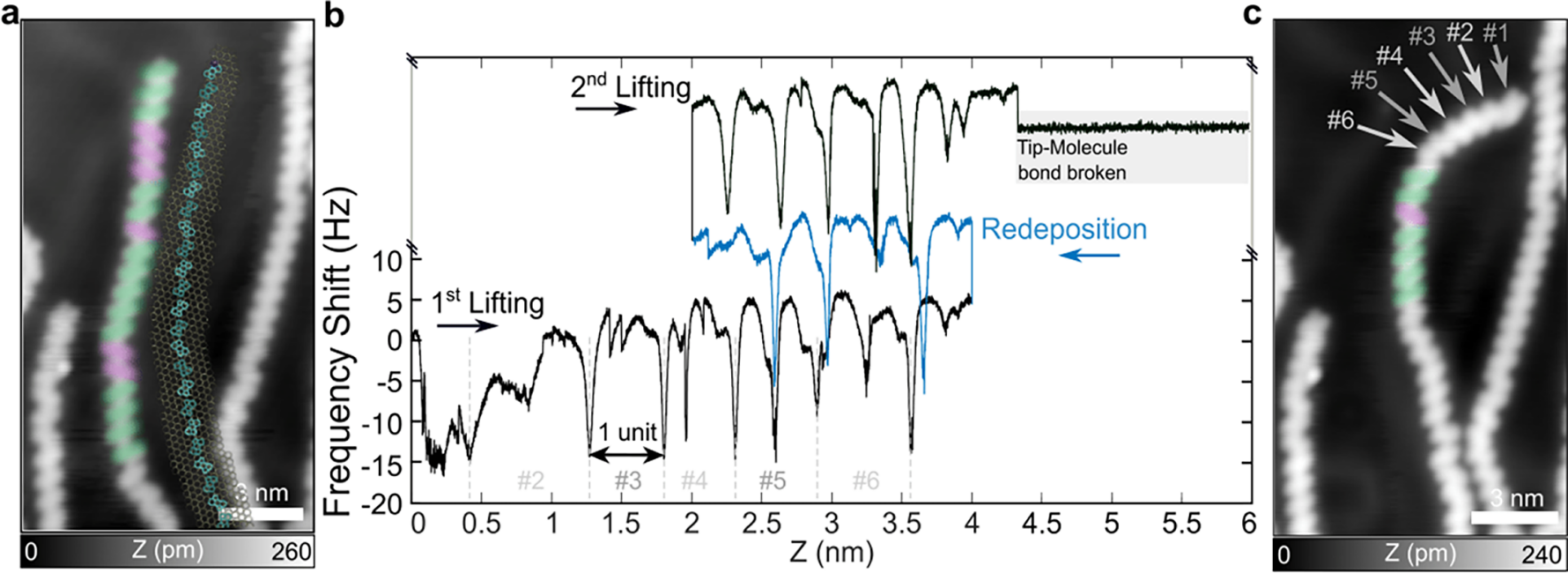
Mechanical response of the CPAA chain during LT-AFM lifting. (a) STM image of the chain before the lifting experiment (*I*_t_ = 1 pA, *V*_s_ = 150 mV). CPAA monomers are marked with green/purple ellipses based on their orientation. The MD simulation overlay shows the atomic adsorption configuration, with gold atoms colored yellow and CPAA carbons in cyan. (b) Frequency shift during the three-step manipulation process, with detachment points of CPAA units marked by gray dashed lines and indexed. (c) STM image of the chain after the lifting experiment, with detached units indicated by arrows and their indices (*I*_t_ = 1 pA, *V*_s_ = 150 mV).

Supporting Movies S2 and S3 provide a detailed picture of the CPAA-chain desorption process. As shown in Figure 3a,e,f, the primary structural change of the chain upon desorption is the loss of planarity. As a given chain monomer detaches from the surface, the steric hindrance between the neighboring units imposes a nonzero torsion angle. This angle depends on the initial on-surface relative orientation (parallel/antiparallel). If consecutive adsorbed units were parallel (ϕ = 0°), then once they detach, they equilibrate at ϕ_1_ = 30°, as shown in Figure 3b—the lowest energy stereoisomer. More interestingly, if consecutive monomers are initially antiparallel (ϕ = 180°), then upon desorption, they equilibrate at ϕ_2_ = 150° (Figure 3b)—a metastable stereoisomer. Moreover, as we continue detaching the molecule, we observe that this metastable conformation is preserved, even after the complete chain desorption (see Figure 3e,f). This has been experimentally confirmed by redepositing the CPAA chain after partial desorption. As shown in Figure 3c, although after manipulation the chain moved slightly toward the lifting axis (as evidenced by the marked reference point), the relative orientation between consecutive units is preserved, even the antiparallel ones. Thus, the hindered rotation around the molecule’s chiral axis (defined by the direction along C–C bonds connecting consecutive monomers), as quantified by the energy barrier at 90° in Figure 1e, stabilizes this class 1 atropisomer in the gas phase.^55^ Consequently, both experimental (Figure 3c) and computational (Figure 3e,f) results confirm the mechanical stability of CPAA metastable conformers during controlled manipulation processes and are expected to persist through multiple rounds of lifting and soft readsorption (i.e., without the molecule snapping away from the tip in an uncontrolled manner). In contrast, when the molecule detaches abruptly from the tip, as at the end of the second lifting shown in Figure 4b, readsorption occurs in an uncontrolled fashion, often resulting in a change in chain conformation. This highlights the metastable nature of these conformers.

### Lifting Surface-Tethered Chains: Sliding Exfoliation vs Delamination

In Figure 4a, we illustrate the initial conformation of a CPAA chain before a lifting event. The total chain length is *L* = 21.8 nm, with its tail tethered to a step edge outside the pictured area. As previously described, to manipulate the chain, the AFM tip is pressed against one end of the chain (yellow dot in Figure 3b,d) to form a stable tip-molecule bond.^8,12,13,33^ Subsequently, by moving the AFM tip away from the surface, the chain is lifted, as depicted in Figure 3a. During this process, the oscillating cantilever supporting the AFM tip is excited at its eigenfrequency *f*_0_, and the frequency shift (Δ*f*) is measured (see Methods). In dynamic AFM, Δ*f* can be directly related to the gradient of the tip–sample interaction force. In this regard, for small oscillation amplitudes (much smaller than the characteristic length scale of the tip–sample interaction force), Δ*f* is approximated to the force gradient using the equation:^56−61^
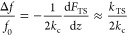
1where *F*_TS_ is the tip–sample interaction force, *k*_c_ is the spring constant of the cantilever, and *z* is the tip–sample distance. This force-gradient interpretation of the frequency shift enables a quantitative understanding of the molecular system’s mechanical response during nanomanipulation.^33^ Consequently, abrupt changes in Δ*f* correspond to sudden changes in the tip–sample interaction force, such as desorption events, including the detachment of individual monomers or the sliding of still-adsorbed molecular segments.^13,17,62,63^

Figure 4b presents the measured frequency shift (Δ*f*) during a representative chain nanomanipulation. Alike curves for other nanomanipulations are provided in Figures S2 and S3 of Section S2 and Figure S4 of Section S3 of the Supporting Information. This specific manipulation process was divided into three stages: (i) first, lifting the chain-end from the surface to a total distance of Δ*z* = 4 nm (black trace labeled as 1st Lifting); (ii) then, redepositing it by displacing the tip toward the surface by Δ*z* = 2 nm (blue trace labeled as Redeposition); and (iii) finally, the exfoliation process continues as the tip is lifted away from the surface until the tip-molecule bond is abruptly broken when the tip–surface distance reaches 4.5 nm (black trace labeled as 2nd Lifting). The conformation of the molecule before and after this process is shown in Figure 4a,c, respectively. In the latter, note how the effects of the manipulation are readily apparent up to the sixth CPAA monomer.

**Figure 5:**
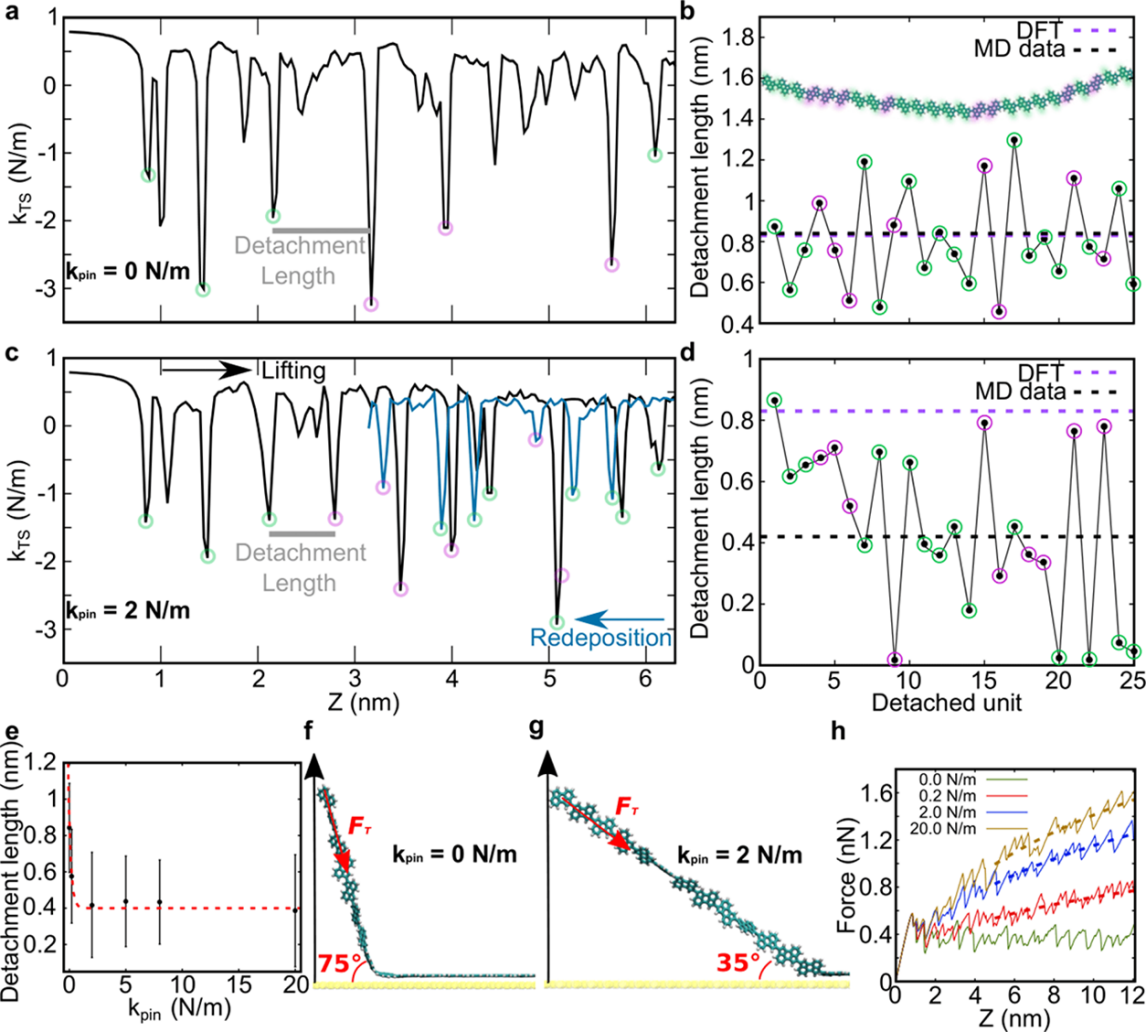
Lifting process with MD simulations. (a) and (c) show the force gradient *k*_TS_ vs tip height during the lifting of a free chain (*k*_pin_ = 0 N/m) and a tethered chain (*k*_pin_ = 2 N/m), respectively. Circles mark monomer desorption/adsorption events and not marked correspond to sliding events (see Section S4 of the Supporting Information). (b) and (d) present the detachment length for each CPAA unit detached from the free and tethered chains, respectively. The dashed purple lines correspond to the size of the monomer obtained from DFT, and the black ones are the average detachment length. (e) shows detachment length as a function of tethering stiffness *k*_pin_, with a red dashed line as a fit to the data. (f) and (g) show chain snapshots at 7 nm height during lifting for different *k*_pin_ values, with red arrows showing the tension force **F**_T_. (h) plots tip force vs height for different *k*_pin_ values, with dashed lines representing a *Z*^1/3^ fit.

Previous studies demonstrated that the periodic modulation in the frequency shift observed in Figure 4b relates to detachment events of consecutive monomers composing alike PAH chains.^13,33^ As the AFM tip steadily moves away from the surface at a constant velocity, one would naturally anticipate an increase in strain within the molecular contact bridging the AFM tip and the surface, resulting in a proportional change in Δ*f* ∝ *k*_eff_. However, this is not the case, as the chain periodically moves toward the lifting axis and/or experiences monomer desorption events. These actions reduce the tension within the molecular contact, leading to abrupt decreases in Δ*f* ∝ *k*_eff_. These dips are clearly visible as repeating patterns in the frequency shift trace, indicated in Figure 4b with gray dashed lines. It should be noted that, aside from these major dips in the Δ*f*, one also observes a weaker modulation of the frequency shift without an apparent periodicity. For instance, during unit #2 detachment, at *z* ≈ 1 nm, one observes an intermediate drop in the recorded frequency shift (see Figure 4b). Although this is discussed in Section S4 of the Supporting Information with atomic details from simulations, it is worth anticipating that such intermediate softening events (i.e., smaller dips in Δ*f* ∝ *k*_eff_ trace) are related to on-surface motion events. That is, during the lifting process, the accumulated tension is released through slip events of the chain as a whole toward the lifting axis, giving rise to a slight reduction in Δ*f*, as also shown for simulations in Figure 5a,c at *z* ≈ 1 nm. Thus, we can differentiate between sliding and desorption events based on their distinct effects on Δ*f*. From our MD simulations, we observed that desorption events are generally marked by a sharp drop in force and a significant decrease in the force gradient, producing large peaks in Δ*f* (see Section S4 of the Supporting Information). In contrast, sliding events only partially alleviate the tension, causing smaller reductions in the force gradient and resulting in peaks of lesser magnitude of Δ*f*. Therefore, the magnitude of the frequency shift peaks serves as a criterion to distinguish between these two phenomena: larger peaks are indicative of desorption, while smaller peaks correspond to sliding.

In Figure 4b, we use dashed lines to indicate the detachment events of single CPAA monomers. Notably, the first and second unit detachments, influenced by the long-range tip–surface interaction, show slight variations in frequency shift traces (see also Figure S4 in the Supporting Information). However, from the third unit onward, detachment events consistently occur at intervals of 0.57 ± 0.06 nm. A close inspection of this detachment length provides unique insights into the mechanical exfoliation of single molecules. First, there is no correlation between detachment length and the relative orientation of consecutive units. For example, the detachment length of unit #3 (see Figure 4a,b), with maximal steric hindrance due to the following unit’s opposed orientation, is similar to that of units #4 and #5, which are followed by units with the same orientation. This contrasts with previous findings on poly-pyrenylene chains, where steric hindrance influenced detachment length by affecting the molecule’s on-surface movement during the sliding exfoliation mechanism.^33^ Second, when comparing detachment length with CPAA monomer size (0.83 nm), we find a significant difference. In previous works, detachment lengths matched the monomer size.^13,33^ This matching is a hallmark feature of a sliding exfoliation mechanism, where the object being lifted freely slides toward the lifting axis as it is pulled. In contrast, the frequency shift modulation associated with CPAA monomer detachment, shown in Figure 4a, is considerably smaller than its size, i.e., ≈0.6 nm as compared to *L*_monomer_ = 0.83 nm. This difference between the nominal length and detachment length is novel and suggests a different mechanism governing the exfoliation process.

To gain an atomically detailed understanding of this novel exfoliation mechanism, we performed all-atom MD simulations. We considered a CPAA chain with an initial configuration identical to the experiments, as shown in the Figure 4a inset. Then, we modeled two distinct desorption processes: one where the chain is free, i.e., only interacting with the surface via nonbonded terms (van der Waals and Coulomb); and a second where, additionally, the chain-end is tethered to the surface. During the lifting process, we computed both the normal load force and its derivative (*k*_TS_), which directly relates to the frequency shift (*k*_TS_ ∝ Δ*f*). In both cases, simulations qualitatively replicate the frequency shift modulation observed experimentally (Figure 5a,c). Most importantly, simulations unequivocally relate sudden *k*_TS_ changes to monomer detachment/readsorption events. When comparing both free and tethered lifting traces, respectively, Figure 5a,c, one important characteristic differentiates them, i.e., the detachment length. In particular, as in previous works,^13,33^ the free chain showed a detachment length comparable to the monomer size (=0.84 nm, see Figure 5b), consistent with a sliding exfoliation process. In contrast, the tethered chain best reproduces the present lifting experiments as it yields a much shorter detachment length ( nm, Figure 5d).

Therefore, the atomistic simulations unveil that the reduced detachment length obtained in experiments (Figure 4b) results from a tethering effect, either to another polymer (as shown in Figure 3b,c) or to a surface defect. In this context, we distinguish for the first time two different exfoliation processes of PAH molecules. The first involves a sliding exfoliation process, where the portion of the molecule still adsorbed slides freely toward the vertical lifting axis, characterized by a detachment length similar to the monomer size. The second corresponds to a delamination process, where one end of the chain is tethered, characterized by a detachment length smaller than the monomer size.

To further explore the influence of tethering on delamination, a series of molecular lifting simulations were performed by varying the stiffness of the tethering point (*k*_pin_). A greater stiffness leads to a marked decrease in the detachment length (see Figure 5e). Specifically, the detachment length decreases from 0.84 nm (the monomer length) to ∼0.4 nm for surface tethering stiffness ranging 0 ≤ *k*_pin_ < 2 N/m. Beyond 2 N/m, the detachment length remains constant at about ∼0.4 nm. Conversely, the surface tethering stiffness can be estimated from the detachment length. For instance, comparing the experimental data in Figure 4b with Figure 5e, one estimates the corresponding tethering stiffness to be ∼0.2 N/m – which is consistent with organometallic bond stiffness values.^64,65^

Another characteristic of delamination is the configuration that the molecule adopts during peeling. Depending on the stiffness, the lifted segment exhibits different angles with respect to the surface. This is shown in Figure 5f,g, where the molecule is lifted at the same height but with a different tethering stiffness. The trend is clear, i.e., the larger the stiffness, the smaller the angle it forms with the surface. The different chain geometry reflects changes in the mechanical energy of the system,^15^ thus playing a critical role in *delamination* dynamics. Specifically, the lifting force, *k*_TS_(*vt* – *z*), is perpendicular to the surface. As the chain resists lifting, it generates a tension (**F**_T_) along its length, opposing the tip’s movement with its vertical component, −|**F**_T_| sin θ. The angle between the lifted chain and the surface is θ (Figure 5f,g). The total vertical force on the end of the molecule, *F*_mol_^*z*^, can be expressed as the sum of these contributions: *F*_mol_^*z*^ = *k*_TS_(*vt* – *z*) – |**F**_T_| sin θ. To desorb a monomer, *F*_mol_^*z*^ must reach a certain value. Then, as θ decreases with stiffer tethering, the opposing force |**F**_T_| sin θ diminishes. This means that the force required to detach a monomer is achieved with smaller tip displacements, leading to shorter detachment lengths, as observed in the experiments and simulations.

A further feature of delamination is the force experienced by the tip during the process of lifting. Our results show that this force increases sublinearly with height, as shown in Figure 5h. Specifically, for heights larger than ∼5 nm, the force scales as *F*_tip_ ∝ *Z*^1/3^, consistent with the peeling model for tethered chains proposed by Silva et al.^15^ Our findings thus confirm this model's predictions, demonstrating its applicability to more complex molecular structures, such as atropisomeric CPAA molecules, besides only graphene ribbons (GNR). Its generality lies in considering only the elastic, adhesion, and tethering properties of the system as the main elements to describe its physical properties. Concomitantly, this agreement emphasizes that the nature of exfoliation processes unveiled in CPAA peeling experiments differs from previous sliding exfoliation works^13,33^ and may be henceforth used as a hallmark feature to identify delamination processes in experiments.

## Conclusions

Here, we provide a comprehensive study, encompassing the synthesis, on-surface alignment, and peeling, of an elusive class of stereochemical isomers, namely, Kemp’s class 1 atropisomers.^51^ Concerning the latter, it also enabled to explore, for the first time, the peeling dynamics of surface-tethered chains, thus unveiling a hallmark feature of *delamination* processes setting it apart from other exfoliation mechanisms.

Concerning the synthesis, our work conclusively demonstrates that, contrary to solution chemistry, on-surface synthesis provides the means to synthesize class 1 sterically frustrated atropisomers. As unveiled through STM and AFM imaging, synthesized CPAA polymers are forced into a planar conformation, which differs from their gas-phase structure. Moreover, the alignment between consecutive monomers is in a racemic mixture of stable and metastable conformers. Interestingly, the specific configurations of consecutive monomers give rise to very distinct alignments of the molecule with respect to the crystallographic directions of the surface where it was adsorbed. Consequently, this results in friction coefficients that depend on the specific stereochemical arrangement of the chain in this molecule–surface contact. In other words, for the same contact area, different friction coefficients are expected.

Following synthesis, the nanomanipulation process unveiled periodic drops in the frequency shift curve during the lifting and redeposition processes of the molecule, which were related to monomer detachment. Importantly, the detachment length does not depend on whether the monomers are arranged in parallel or antiparallel configurations. Moreover, unlike previous works,^13,33^ where the detachment length matched the monomer size, our results reveal a detachment length that is smaller than the monomer size. This allowed distinguishing, for the first time, two different exfoliation processes of PAH molecules: sliding exfoliation and delamination. Additionally, our results show that the force exerted on the tip during delamination aligns well with the analytical model proposed by Silva et al. for peeling of tethered chains.^15^

## Methods

### Sample Preparation

The surface of a gold Au(111) single crystal purchased from Mateck GmbH was cleaned by several sputtering and annealing cycles in ultrahigh vacuum (UHV). 2,7-Dibromo-CPAA (see Figure 1a) was thermally evaporated at ∼380 K onto the gold substrate kept at room temperature.^66^ The evaporation rate was checked using a quartz microbalance. The Ullmann coupling reaction of the precursors was activated by annealing the substrate first at ∼470 K and then above 500 K for removing the dissociated Br atoms.

### STM/AFM Experiments

STM/AFM experiments were carried out at 4.8 K with an Omicron GmbH low-temperature apparatus operated with Nanonis RC5 electronics. Constant-height AFM images were acquired with CO-terminated tips using a commercial tuning fork sensor (quality factor *Q* = 10,000–25,000, nominal spring constant *k*_c_ = 1800 N m^–1^) in the qPlus configuration 3 driven on resonance at a small constant tip oscillation amplitude *A* = 50 pm. The recorded frequency shift is then Δ*f* = 0.5*f*_0_*k*_TS_/*k*_c_, where *f*_0_ = 26 kHz is the unperturbed resonance frequency, and *k*_TS_ = d*F*/d*Z* is the gradient of the vertical force exerted by the tip at a distance *Z* from the surface. Constant-current STM images were acquired with *A* = 0 after sharpening the tungsten tip at the end of the free fork by gentle indentation into the gold. All voltages refer to the sample bias with respect to the tip.

### Experimental Lifting

Pulling experiments were performed with oscillating gold-decorated tips, while simultaneously recording the tunneling current at a typical bias of 40 μV. A single CPAA chain molecule identified by STM was picked up by slowly approaching the AFM tip to the molecule at one of its extremities. Attachment of the molecule to the tip apex was observed as an abrupt jump in the frequency shift and current signals. So-called force spectroscopic measurements were performed upon retraction at a constant velocity of 50 pm s^–1^. The relation between Δ*f* and *k*_TS_ remains valid, but the effective compliance 1/*k*_TS_ is now a sum of contributions from the lifted chain segment and its interactions with the tip, the surface, and the still-adsorbed segment. The observed Δ*f* maxima are rounded, whereas the sharp dips associated with abrupt monomer detachments are slightly broadened mainly for instrumental reasons. The resonance-tracking and oscillation-control electronic circuits cannot properly follow the underlying force jumps over a range [−*A*, +*A*] about the appropriate *Z*-values.

### Quantum Mechanical (QM) Calculations

All QM calculations were performed using the Gaussian09 suite of programs. A full geometry optimization of a trimer of CPAA was carried out with the B3LYP density functional and the Dunning’s correlation-consistent cc-pVDZ basis set. For this optimized conformation, the Hessian matrix and the corresponding vibrational normal modes were calculated at the same level of accuracy. Additionally, total energies of optimized conformations at fixed opposite dihedral angles (from 0° to 180° in steps of 5°) between the planes of the central and two terminal CPAA units were computed. These results were then used to build a QM-derived CPAA intramolecular force-field.

### Molecular Dynamics (MD) Simulation Details

MD simulations were carried out using the GROM- ACS-2018.2(67) simulation package in a hybrid GPU–CPU computing architecture.^68^ All simulations were performed in the canonical (*NVT*) ensemble, where the number of atoms (*N*), volume (*V*), and temperature (*T* = 5 K) were held constant. The simulation parameters and algorithms used are consistent with the GolP force-field,^69^ namely, periodic boundary conditions, smooth particle mesh Ewald summation^70^ with cubic spline interpolation of the electrostatic energy contribution, and a real-space Coulombic cutoff of 1 nm. Interatomic CPAA-gold van der Waals interactions were also truncated at 1 nm. Previously, these parameters allowed obtaining a quantitative agreement with experiments on both adsorption geometries and lifting contact stiffness.^8,19,33,71,72^ The *Z* dimension of the simulation box surpassed the system size in the *X* and *Y* directions so that spurious contributions from periodic images were avoided, even during lifting simulations. A Langevin thermostat^73^ was employed to bring the CPAA monomers' mean temperature to 5 K after very rapid detachment or slip events. A damping rate of 1 ps^–1^ ensured that any excess heat was soon dissipated between such events. Separately thermostatting reorientable dipoles attached to the fixed gold atoms, as in ref (69), allows to accurately account for the polarization of Au(111) by moving partial atomic charges. Equations of motion were integrated with an accurate and efficient leapfrog stochastic dynamics integrator^73^ with a time step of 0.5 fs. The small integration step allowed for the fast dynamics of the hydrogen atoms. Coordinates, forces, and velocities of each moving atom were written every 2 ps, while the force applied during the lifting process was written every 0.025 ps.

### Atomic Level Models and Force-Fields

We considered an unreconstructed Au(111) slab with five atomic layers parallel to the *XY* plane and perpendicular to the *Z* axis. The *X*-axis was along the [112̅] crystallographic direction of bulk Au. The simulated polymers ranged from 10 to 30 units of CPAA chains, initially placed with all units parallel to the Au(111) surface at a distance of 0.45 nm. We considered different configurations of the molecular chains, where the contiguous monomer units can be either parallel or antiparallel. The interactions between the atoms composing the chain were modeled via a QM-derived intramolecular force-field purposely generated for CPAA using the Joyce methodology^74^ and code.^75^ This methodology to parametrize the force-field has shown excellent agreement with structural and dynamics properties measured in experiments,^19,33,76−79^ and further information can be found in Section S1 of the Supporting Information. An additional validation of the accuracy of this methodology is demonstrated in Figure 1e, where the energy barrier for the rotation of the aryl–aryl bond between two units, as predicted by classical simulations, closely matches the results from DFT calculations. The interaction between the Au(111) atoms and the chain atoms was described by the QM-derived GolP force-field,^69^ using parameters purposely adjusted to describe the interaction between Au(111) and aromatic compounds.

### Simulation Protocol

For all chain sizes and configurations considered, the equilibrium adsorption configuration was obtained by quenching the temperature from 450 to 5 K at a cooling rate of 0.0345 K/ps. The system was then equilibrated over an additional 1 ns at *T* = 5 K. For these starting equilibrium configurations, we manipulated the molecule. Further details on the protocol and agreement with experiments are provided in prior works.^19,33^

We lift/deposit the chain from the surface by connecting a point-like ‘tip atom’ to the para-axis carbon atom of the first CPAA unit via a vertical spring of stiffness *k*_TS_ = 0.23 N/m, representing the tip-molecule bond after successful pickup. For this, we use the steered molecular dynamics (SMD) technique, where the ‘tip atom’ can only move in a vertical (*Z* axis) manner. The velocity at which the ‘tip atom’ is lifted/deposited from the surface was 1.0 m/s. The frequency shift as a function of height is then obtained from the gradient of the normal force (*Z* axis) recorded while lifting/depositing the chain.

In the cases where we consider the effect of the chain being tethered, we apply a harmonic constraint with stiffness *k*_pin_ to the para-axis carbon atom of the last unit (relative to the unit that is initially lifted) of the CPAA molecule. In this regard, we consider different stiffnesses *k*_pin_ for the force constant associated with the harmonic potential.

## Supporting Information Available

Parametrization and validation of the CPAA QMD-FF; additional MD simulations of CPAA adsorption conformations and lifting processes; MD adsorption configurations and lifting processes of CPAA molecules; MD adsorption configurations and lifting processes of additional CPAA molecules; additional exfoliation experiments of CPAA chains; additional exfoliation experiments of CPAA molecules; force and force gradient analysis: detachment and sliding events; and detachment and sliding events (PDF)
The ZIP is available free of charge at https://pubs.acs.org/doi/10.1021/zzz, which contains the Supplementary Movies S1–S3. Quenching process and lifting process.(ZIP)
