# Supplementary material for: On-Surface Synthesis and Cryogenic Exfoliation of Sterically Frustrated Atropisomers
Source: ACS Nano. 2025 Apr 1;19(14):13805–16. doi: 10.1021/acsnano.4c16645 (PMC12005049; doi:10.1021/acsnano.4c16645)
Supplement: Supplementary file 1 — nn4c16645_si_001.pdf [file nn4c16645_si_001.pdf]

Supporting Information: On-Surface Synthesis and Cryogenic Exfoliation of Sterically Frustrated Atropisomers

Philipp D'Astolfo,[†,||] J.G. Vilhena,[*,‡,||] Simon Rothenbühler,[¶] Carl Drechsel,[†] Oscar Gutiérrez-Varela,[‡] Robert Häner,[¶] Silvio Decurtins,[¶] Shi-Xia Liu,[*,¶] Giacomo Prampolini,[§] Rémy Pawlak,[*,†] and Ernst Meyer[*,†]

†*Department of Physics, University of Basel, Klingelbergstrasse 82, 4056 Basel, Switzerland*

‡*Instituto de Ciencia de Materiales de Madrid (ICMM), CSIC, Madrid 28049, Spain*

¶*Department of Chemistry, Biochemistry and Pharmaceutical Sciences, W. Inäbnit Laboratory for Molecular Quantum Materials, University of Bern, Freiestrasse 3, Bern, CH 3012 Switzerland*

§*CNR–Consiglio Nazionale delle Ricerche, Istituto di Chimica dei Composti Organo Metallici (ICCOM-CNR), Area della Ricerca, via G. Moruzzi 1, I-56124 Pisa, Italy*

||*equally contributing*

E-mail: guilherme.vilhena@csic.es; shi-xia.liu@unibe.ch; remy.pawlak@unibas.ch; Ernst.Meyer@unibas.ch

This supporting information document includes the following:

- S1. Force field parameterization and validation
- S2. Additional MD Simulations of CPAA Adsorption Conformations and Lifting Processes
- S3. Additional Exfoliation Experiments of CPAA chains
- S4. Force and Force Gradient Analysis: Detachment and Sliding Events
- S5. Captions for Supplementary Movie S1: Quenching Process
- S6. Captions for Supplementary Movies S2 and S3: Lifting Process

S1. Force field parameterization and validation

The total energy of the CPAA + Au(111) system is described as a sum of two terms accounting for the energy cost of polymer deformations with respect to the reference (gas-phase) configuration and for the interactions of CPAA atoms with the gold substrate. The intra-molecular contribution $E^{\mathrm{FF}}_{\mathrm{intra}}$ depends on a joint set of internal coordinates, namely bond lengths $\{b\}$, bond angles $\{\theta\}$, relatively stiff $\{\varphi\}$ or compliant $\{\phi\}$ dihedral angles between pairs, triplets and quadruplets of neighboring bonded atoms, respectively.

The corresponding deformation energies are modeled as sums over independent set elements, N_b, N_θ , N_φ, N_ϕ being their respective numbers. The first three sums involving stiff coordinates (in particular improper φ's describing deviations from planarity of the pyrenylene units and out-of-plane bending about their connecting C-C bonds) are modeled as quadratic functions of the deviations from their equilibrium values $\{b_0\}$, $\{\theta_0\}$, $\{\varphi_0\}$ with respective force-constant sets $\{k_b\}$, $\{k_\theta\}$ and $\{k_\varphi\}$. To account for twists $\{\phi\}$ about C-C bonds, which are expected to show large deviations, e.g. upon chain adsorption, N_ϕ Fourier cosine series truncated beyond m terms are used instead, multiplied by respective torsional force constants $\{k_\phi\}$. The parameters sets $\{k_b\}$, $\{k_\theta\}$, $\{k_\varphi\}$, $\{k_\phi\}$ and $\{b_0\}$, $\{\theta_0\}$, $\{\varphi_0\}$, $\{\phi_{m0}\}$ are automatically determined by the JOYCE package[1,2]

applied to a reference molecule for which QM data have been purposely computed as described in Methods. Note that JOYCE is a well established, freely available method to derive accurate force fields from Quantum-Mechanical data.[1] To reduce the computational burden without losing the required accuracy, the parameterization was carried out on the CPAA trimer shown in Supporting Figure S1d inset, which contains all types of bonds also present in the longer polymer. Because the total number of FF parameters exceeds the number of independent degrees of freedom ($3N$-5 for linear chains), the parametrization is achieved through a least-squares minimization of the objective function:

$$I^{\mathrm{intra}} = W\left[U - E^{\mathrm{FF}}_{\mathrm{intra}}\right]^2 + \sum_{K\leq L}^{3N-6} \frac{2W''_{\mathrm{KL}}}{(3N-6)(3N-5)}\left[H_{\mathrm{KL}} - \left(\frac{\partial^2 E^{\mathrm{FF}}_{\mathrm{intra}}}{\partial Q_{\mathrm{K}}\partial Q_{\mathrm{L}}}\right)\right]^2 \tag{1}$$

where U is the QM computed energy of the trimer, Q_{K} is the $(K-th)$ the normal mode coordinate, the QM Hessian matrix H_{KL} in the (Q_{K}, Q_{L}) representation and its FF counterpart, all evaluated in the gas-phase equilibrium conformation. W and $W"_{\mathrm{KL}}$ are weighting factors determined on the basis of experience with various molecules.[2] The optimized FF parameters obtained for the end and central trimer units are adopted for the end and all internal units of the CPAA chain in all subsequent simulations.

The accuracy of the derived intra-molecular force field is first validated by comparing QM and FF results, see Figure S1. In a first instance, Figures S1a-and-b show that the structure predicted by the QMD-FF is indistinguishable from the predicted by QM data. Moreover, the agreement between vibration mode eigenvectors and frequencies obtained (top and bottom panels of Supporting Figure S1c) is also remarkable. Finally, the parametrized torsional energy profile shown in the bottom panel of Supporting Figure S1d also shows an excellent agreement between the QM and QMD-FF data for flexible CPAA coordinates.

The polymer-gold interaction energy $E^{\mathrm{FF}}_{\mathrm{inter}}$ is described by pairwise sums of 12-6 Lennard-Jones (L-J) and Coulomb potentials between all atoms $\{i\}$ of the CPAA chain and all interaction sites $\{j\}$ of the Au(111) slab specified while testing the GolP FF,[3] namely hollow surface sites for

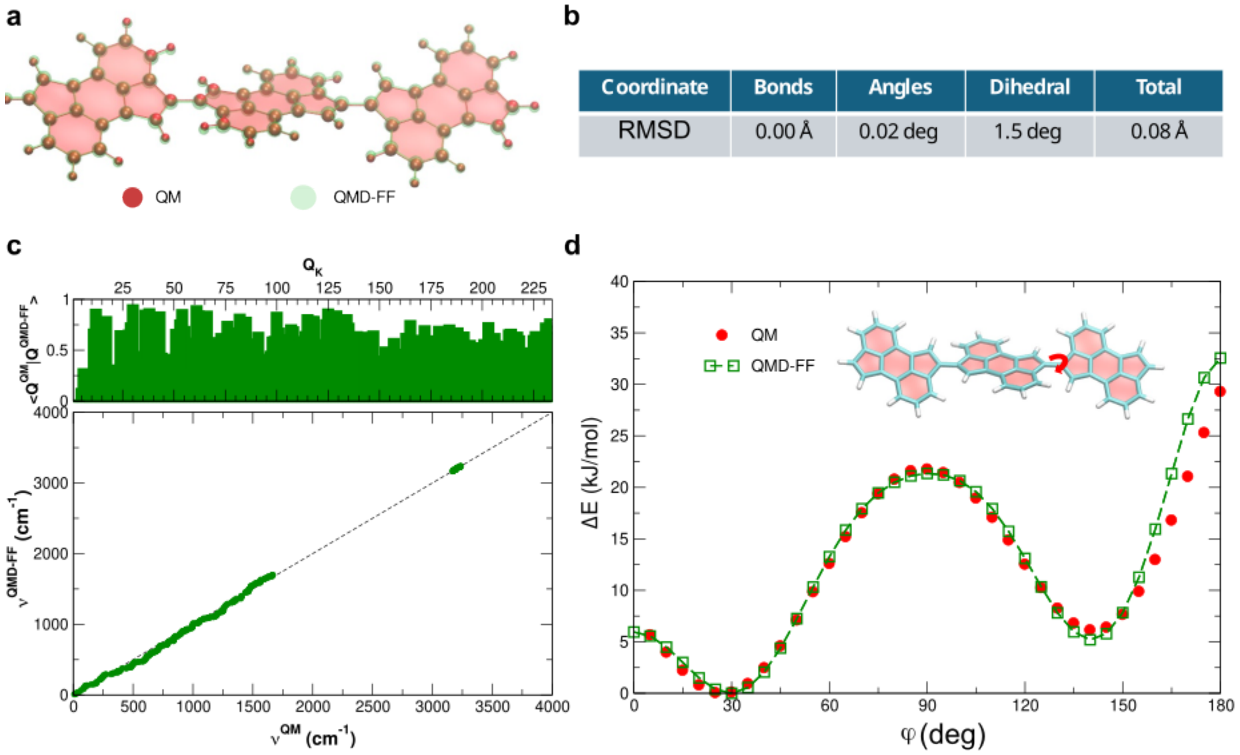

Coordinate	Bonds	Angles	Dihedral	Total
RMSD	0.00 Å	0.02 deg	1.5 deg	0.08 Å

Figure S1: **Validation test of the CPAA QMD-FF.** **(a)** overlap of the QM (full red spheres) and QMD-FF (transparent green spheres) optimized structure; **(b)** RMSD on the internal coordinates of the QMD-FF optimized geometry with respect to thew reference QM; **(c)** Comparison of the QM and QMD-FF description on a local harmonic approximation: top, normal mode overlap and bottom, vibrational frequency correlation plot; **(d)** Comparison of the relaxed torsional profiles for the flexible dihedral phi.

L-J interactions and dipoles at all atomic sites for Coulomb interactions[3] L-J strength and range parameters for C and H atoms were taken from an improved fit against more accurate VdW-DFT QM calculations.[4] To be consistent with GolP, L-J parameters, as well as partial atomic charges for CPAA were taken from the OPLS-AA FF[5,6] and combined with the L-J parameters for Au(111) via standard mixing rules. Note that the GolP parametrization differs substantially from a plain atom-centered L-J potential.[3] Additionally, we previously bench-marked the interaction energy between an alike PAH polymer and Au(111).[7,8] Not only the adsorption energies are in agreement with reference QM data,[7] but also this potential (GolP+Joyce) is capable of describing in quantitative agreement the interaction stiffness during both desorption[7] as well as sliding[8] dynamics of a PAH on surface – a fact that was further corroborated in the present work as shown in Figures 2, 4 and 5.

S2. Additional MD Simulations of CPAA Adsorption Conformations and Lifting Processes

Figures S2a, S2f, S3a, and S3d show the alignment on the Au(111) surface of different CPAA molecular chains with different parallel/antiparallel arrangements, after annealing at 450 K and subsequent cooling to 5 K (see Methods in the main text). Monomers are colored differently based on their parallel or antiparallel orientation for clarity. Note that in all cases, the alignment is different on the surface.

Figures S2b, S2d, S2g, S2i, S3b, and S3e show graphs of the force gradient as a function of height for lifting simulations of different CPAA molecular chains considering various tetherings. For more detailed information of each graph, we refer to the captions of Figures S2 and S3. As observed in all the figures, regardless of the molecule's size or the parallel/antiparallel configuration of its monomers, the force gradient exhibits drops associated with unit desorption events, which are indicated by vertical gray dashed lines.

Figures S2c, S2e, S2h, S2j, S3c, and S3f show the detachment length as a function of the detached unit for the different lifting simulations of the various CPAA molecular chains presented in this section. For further details of each graph, we refer to the captions of Figures S2 and S3. It is important to note that in all cases, the detachment length shows to be independent of the parallel or antiparallel arrangement of contiguous monomers. However, we observe that when comparing the detachment length of free chains with that of tethered chains, it decreases in the presence of tethering, as seen in Figures S2c, S2e, S2h, S2j, S3c, and S3f, and as discussed in the main text.

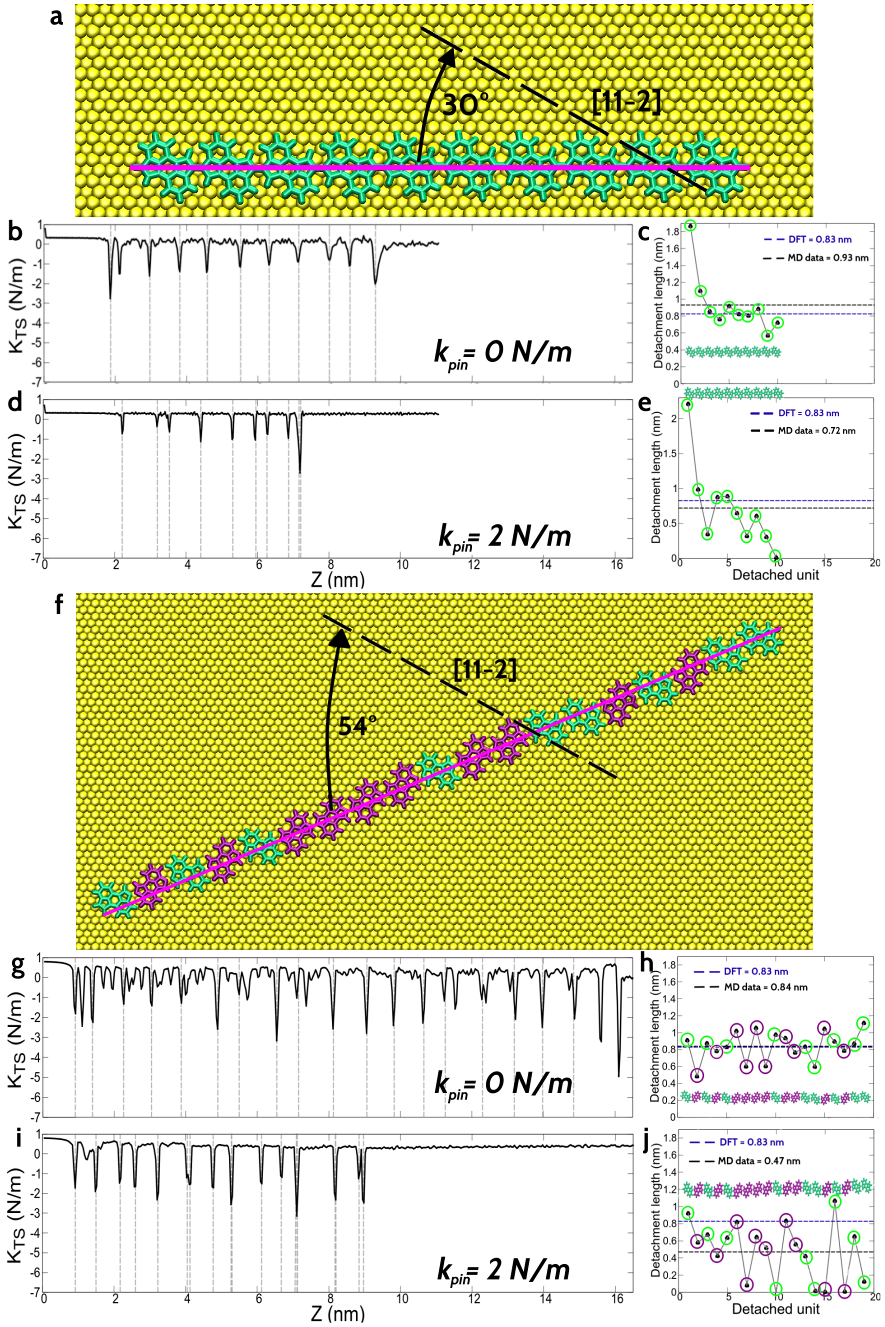

Figure S2: **MD Adsorption Configurations and Lifting Processes of CPAA Molecules.** **(a)** shows the alignment of a CPAA molecular chain with 10 parallel units on the Au(111) surface after annealing at 450 K and cooling to 5 K using MD. **(b)** and **(d)** show the force gradient vs height during lifting of the chain in (a) for cases without tethering and with a tethering stiffness of 2 N/m, respectively. Vertical gray dashed lines mark unit desorption events. **(c)** and **(e)** show the detachment length vs detached unit corresponding to (b) and (d), respectively. **(f)**, **(g)**, **(i)**, **(h)**, and **(j)** show the same analysis as above, but for a 19-unit molecular chain shown in (f).

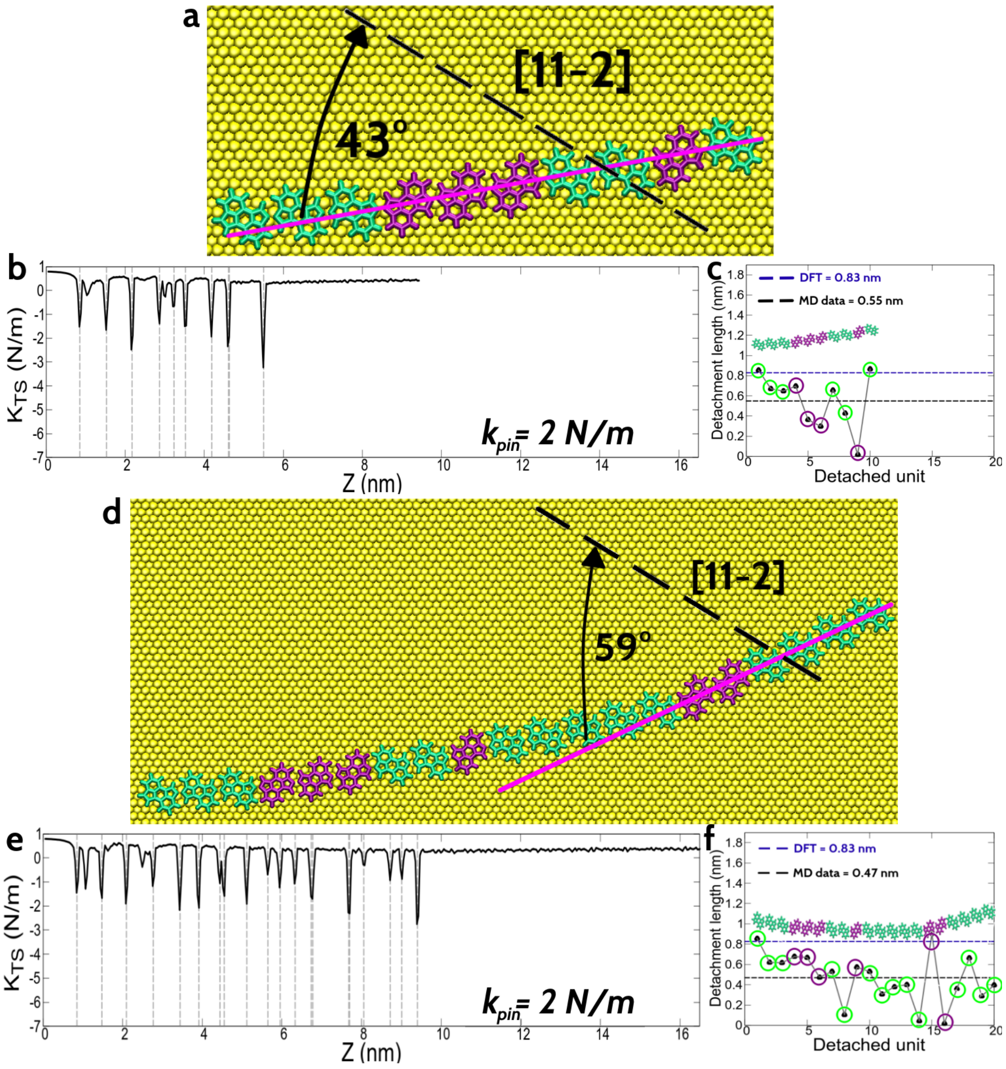

Figure S3: **MD Adsorption Configurations and Lifting Processes of Additional CPAA Molecules.** **(a)** and **(d)** show the alignment of CPAA molecular chains with 10 and 20 units, respectively, on the Au(111) surface after annealing at 450 K and cooling to 5 K using MD. The first 10 units in both molecules have the same parallel/antiparallel order from left to right. **(b)** and **(e)** show the force gradient as a function of height during lifting of the chains in (a) and (d), respectively, with a tethering stiffness of 2 N/m. Vertical gray dashed lines mark unit desorption events. **(c)** and **(f)** show the detachment length vs detached unit for the data in (b) and (e), respectively.

S3. Additional Exfoliation Experiments of CPAA chains

Figures S4a, S4d, and S4g show STM images before AFM exfoliation experiments of different CPAA molecules, while the Figures in S4c, S4f, and S4i show STM images after the exfoliation. The lengths of the manipulated chains are 7.9 nm, 8.3 nm, and 13.2 nm for Figures S4a, S4d, and S4g, respectively. In all cases, the STM images before and after exfoliation clearly demonstrate the molecular chain nanomanipulation by showing that the molecules are in different positions.

Figures S4b, S4e, and S4h show graphs of the frequency shift as a function of tip height in the AFM exfoliation experiments. The experimental parameters and protocols are the same as those used for the results presented in Figure 4 of the main text. Similar to what is observed there, all the graphs presented here exhibit pronounced drops in the frequency shift periodically, which are associated with events of molecular unit desorption, and a drop to zero in the frequency shift when the tip-sample bond breaks. The gray dashed vertical lines represent positions in tip height associated with monomer desorption events. The average detachment lengths associated with each experiment are 0.69 nm, 0.56 nm, and 0.53 nm for the data presented in Figures S4b, S4e, and S4h, respectively. As observed in the data in Figure 4b of the main text, the detachment lengths obtained in the figures presented here smaller than the size of a monomer, 0.83 nm. Another observation regarding the similarity of the data presented here with the data from the main text is that for the desorption of the first two units, the graph of the frequency shift as a function of tip height shows the influence of long-range tip-surface interactions, showing as slight variations in the frequency shift traces.

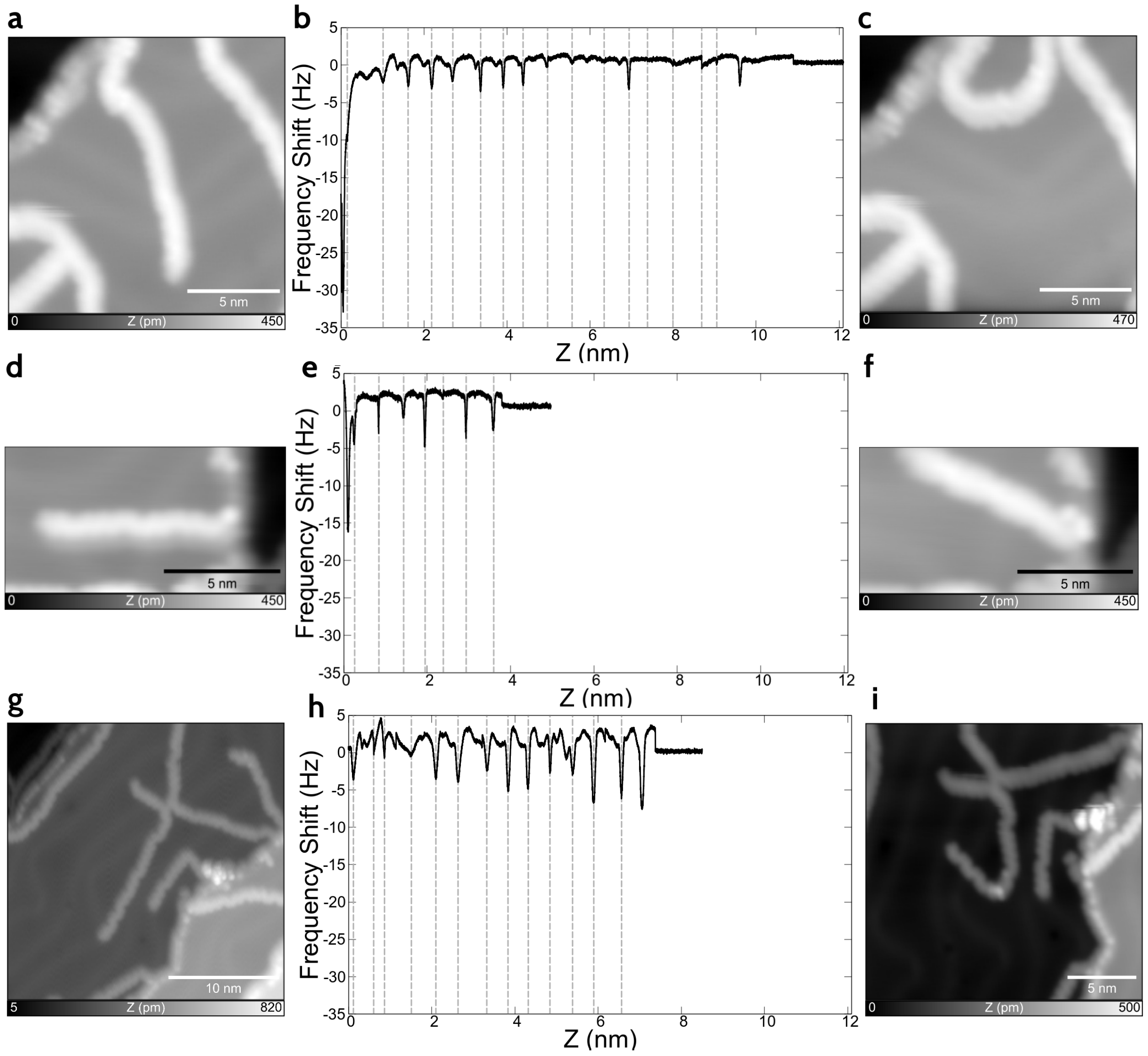

Figure S4: **Additional Exfoliation Experiments of CPAA molecules.** **(a)**, **(d)**, and **(g)** show STM images ($I_\mathrm{t} = 1$ pA, $V_\mathrm{s} = 150$ mV) of different CPAA molecular chains before nanomanipulation. **(b)**, **(e)**, and **(h)** show the frequency shift as a function of tip height for AFM exfoliation experiments with the different CPAA molecular chains shown in (a), (d), and (g), respectively. The gray dashed vertical lines indicate tip heights corresponding to monomer desorption events. **(c)**, **(f)**, and **(i)** show STM images ($I_\mathrm{t} = 1$ pA, $V_\mathrm{s} = 150$ mV) of the molecular chains after nanomanipulation for the experiments in (b), (e), and (h), respectively.

S4. Force and Force Gradient Analysis: Detachment and Sliding Events

Figure S5a shows the force gradient and S5b the force for a CPAA molecular chain lifting process. The chain considered is the same as the one shown in the inset of Figure 4a in the main text, and the tethering effect is accounted for with a force constant of $k_{\mathrm{pin}} = 0.2$ N/m. Purple circles highlight CPAA unit desorption events. The force profile exhibits a sawtooth-like behavior characteristic of a stick-slip process. During desorption events, force accumulates until the CPAA unit detaches, releasing tension and causing the force value to drop. These drops in force value due to desorption processes are reflected as drops in the force gradient, similar to what is observed in experimental data with the frequency shift, which is proportional to the force gradient. However, tension is not only released during desorption events, as seen in the force drops and force gradient drops that are not circled. Drops in force unrelated to desorption events occur due to the sliding of CPAA units still on the surface. Then, both events (desorption and sliding), can be distinguished by analyzing the force gradient. Generally, the drops in the force gradient are more pronounced during desorption events, while sliding events result in smaller changes. This distinction can be quantified by calculating the average magnitude of the force gradient for both types of events. For the data shown in Figure S5a, the average magnitude for desorption events is 1.45 $\pm$ 0.36 N/m, while for sliding events, it is 0.62 $\pm$ 0.44 N/m. Therefore, larger drops in the frequency shift, which is directly proportional to the force gradient, are associated with desorption, and smaller drops are linked to sliding events.

The second drop in the force gradient in Figure S5a and in Figures 5a and 5c of the main text is noteworthy. As seen in each of the figures, this drop in the force gradient is not associated with a desorption event but with a sliding event. Figure S5c shows different snapshots of the detachment-sliding-detachment process corresponding to the first three drops in the force gradient presented in Figure S5a. What is observed is that after detaching the first CPAA unit from the surface, the chain slides toward the vertical axis where the molecule is being lifted. This can be seen in the

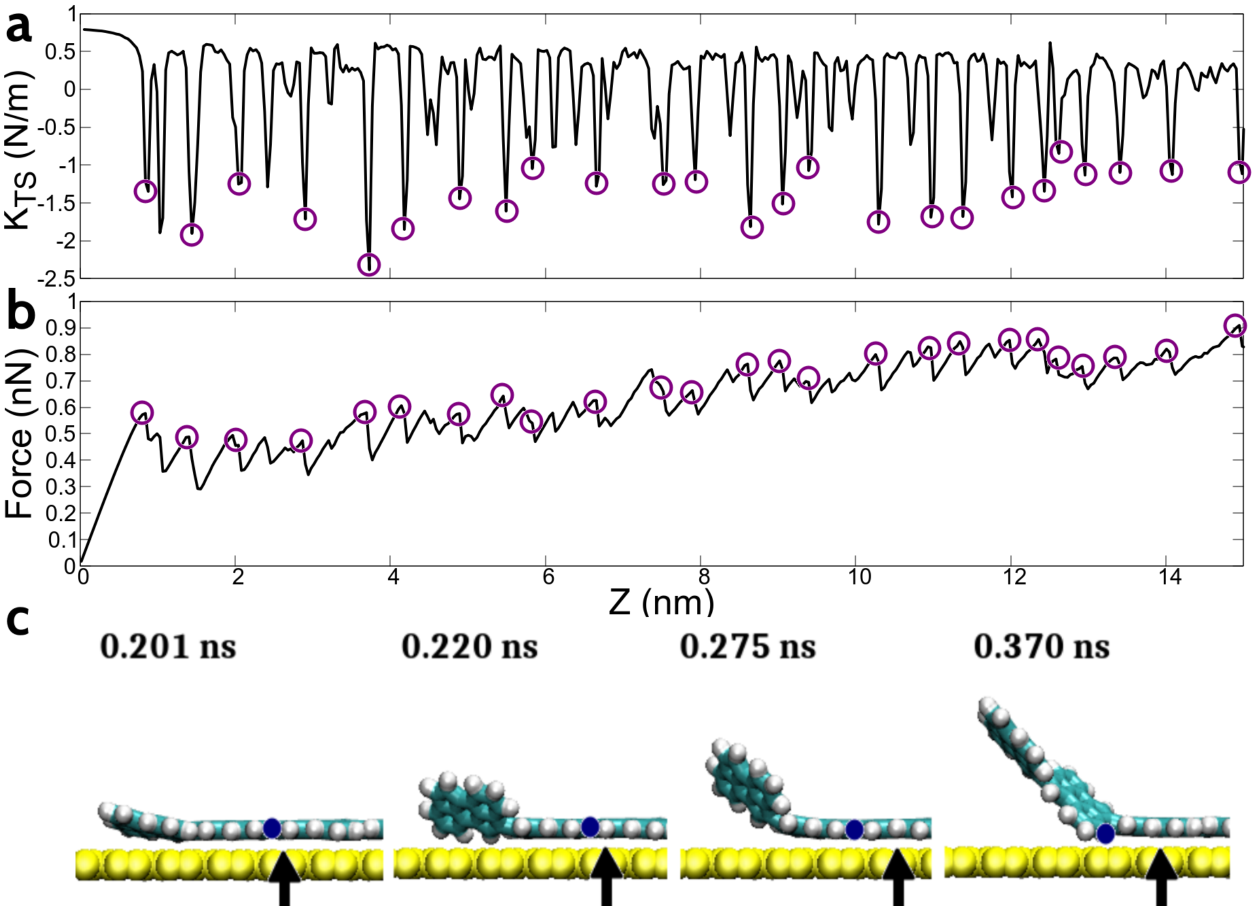

Figure S5: **Detachment and Sliding Events.** **(a)** shows MD data of the force gradient, and **(b)** shows the force as a function of height for a CPAA molecular chain, as the one shown in the inset of Figure 4a in the main text. Tethering is considered with a force constant of $k_{pin} = 0.2$ N/m. Purple circles indicate CPAA unit desorption events. **(c)** shows snapshots of the detachment-sliding-detachment process corresponding to the first three drops in the force gradient of (a). Each image has its time-stamped. The blue circle marks a hydrogen atom of the molecular chain, and the black arrow indicates a fixed position in the surface, highlighting the chain's sliding during the intermediate step between the two detachments, associated with the second drop in (a).

atom marked with the blue circle, which changes its position between the images corresponding to the simulation times of 0.220 ns and 0.275 ns. The drop in the force gradient has a magnitude comparable to a desorption event, and even greater in some cases.

The behavior is also present in the experiments, as observed in Figure 4b of the main text at a height of ~ 1 nm, between the detachment of the 1st and 2nd CPAA units, in agreement with the MD data. Therefore, this characteristic shows to be general in the lifting process of this molecular chain.

S5. Captions for Supplementary Movie S1: Quenching Process

Supporting Movie S1 shows an MD simulation of the quenching process for a 12-unit CPAA molecule, cooling from 450 K to 5 K over 5 ns. The arrangement between the molecule's monomers exhibits both parallel and antiparallel configurations. During quenching, the CPAA molecule undergoes rapid diffusion due to the high initial temperature, exploring various alignment configurations relative to the crystallographic directions of the Au(111) surface. As the system cools, the molecule eventually adopts its equilibrium alignment on the surface.

S6. Captions for Supplementary Movies S2 and S3: Lifting Process

Supporting Movie S2 shows the MD simulation of the lifting process for a CPAA molecule without tethering, while Supporting Movie S3 shows the lifting process for a CPAA molecule with tethering, considering a stiffness of 2 N/m. As observed, the molecule loses planarity in the desorbed segment due to steric hindrance between neighboring monomers; however, it maintains this angle between monomers throughout the entire lifting process. The specific angle depends on the initial on-surface relative arrangement between contiguous monomers, whether parallel or antiparallel, as detailed in the main text. Additionally, Movie S2 shows how the molecule's still-adsorbed segment slides toward the vertical axis of lifting, a characteristic movement of a *sliding exfoliation* process. On the other hand, Movie S3 shows that the still-adsorbed segment exhibits reduced mobility compared to the CPAA molecule in Movie S2, a characteristic movement of a *delamination* process.

References

1. https://joyce-documentation.gitlab.io/.

2. Barone, V.; Cacelli, I.; De Mitri, N.; Licari, D.; Monti, S.; Prampolini, G. Joyce and Ulysses: integrated and user-friendly tools for the parameterization of intramolecular force fields from quantum mechanical data. Phys. Chem. Chem. Phys. **2013**, 15, 3736–3751.

3. F. Iori, R. Di Felice, E. Molinari, S. C. GolP: An Atomistic Force-Field to Describe the Interaction of Proteins With Au(111) Surfaces in WaterGolP: An Atomistic Force-Field to Describe the Interaction of Proteins With Au(111) Surfaces in Water. J. Comput. Chem. **2009**, 30, 1545–1614.

4. Wright, L. B.; Rodger, P. M.; Corni, S.; Walsh, T. R. GolP-CHARMM: First-principles based force fields for the interaction of proteins with Au(111) and Au(100). J. Chem. Theory Comp. **2013**, 9, 1616–1630.

5. Jorgensen, W. L.; Maxwell, D. S.; Tirado-rives, J. Development and Testing of the OPLS All-Atom Force Field on Conformational Energetics and Properties of Organic Liquids. J. Am. Chem. Soc. **1996**, 118, 11225–11236.

6. Damm, W.; Frontera, A.; Tirado-Rives, J.; Jorgensen, W. L. OPLS all-atom force field for carbohydrates. J. Comput. Chem. **1997**, 18, 1955–1970.

7. Pawlak, R.; Vilhena, J. G.; D'astolfo, P.; Liu, X.; Prampolini, G.; Meier, T.; Glatzel, T.; Lemkul, J. A.; Häner, R.; Decurtins, S.; Baratoff, A.; Pérez, R.; Liu, S. X.; Meyer, E. Sequential Bending and Twisting around C-C Single Bonds by Mechanical Lifting of a Pre-Adsorbed Polymer. Nano Letters **2020**, 20, 652–657.

8. Vilhena, J. G.; Pawlak, R.; D'Astolfo, P.; Liu, X.; Gnecco, E.; Kisiel, M.; Glatzel, T.; Pérez, R.; Häner, R.; Decurtins, S.; Baratoff, A.; Prampolini, G.; Liu, S. X.; Meyer, E. Flexible Super-

lubricity Unveiled in Sidewinding Motion of Individual Polymeric Chains. Physical Review Letters **2022**, 128, 1–6.
